# Swin-Net: A Swin-Transformer-Based Network Combing with Multi-Scale Features for Segmentation of Breast Tumor Ultrasound Images

**DOI:** 10.3390/diagnostics14030269

**Published:** 2024-01-26

**Authors:** Chengzhang Zhu, Xian Chai, Yalong Xiao, Xu Liu, Renmao Zhang, Zhangzheng Yang, Zhiyuan Wang

**Affiliations:** 1 School of Humanities, Central South University, Changsha 410012, China; anandawork@126.com; 2 School of Computer Science and Engineering, Central South University, Changsha 410083, China; 214712177@csu.edu.cn (X.C.); 224712218@csu.edu.cn (R.Z.); 224712205@csu.edu.cn (Z.Y.); 3 Department of Medical Ultrasound, Hunan Cancer Hospital/The Affiliated Cancer Hospital of Xiangya School of Medicine, Central South University, Changsha 410031, China; liuxu@hnca.org.cn

**Keywords:** swin-transformer, medical image segmentation, breast tumor, ultrasonic image segmentation

## Abstract

Breast cancer is one of the most common cancers in the world, especially among women. Breast tumor segmentation is a key step in the identification and localization of the breast tumor region, which has important clinical significance. Inspired by the swin-transformer model with powerful global modeling ability, we propose a semantic segmentation framework named Swin-Net for breast ultrasound images, which combines Transformer and Convolutional Neural Networks (CNNs) to effectively improve the accuracy of breast ultrasound segmentation. Firstly, our model utilizes a swin-transformer encoder with stronger learning ability, which can extract features of images more precisely. In addition, two new modules are introduced in our method, including the feature refinement and enhancement module (RLM) and the hierarchical multi-scale feature fusion module (HFM), given that the influence of ultrasonic image acquisition methods and the characteristics of tumor lesions is difficult to capture. Among them, the RLM module is used to further refine and enhance the feature map learned by the transformer encoder. The HFM module is used to process multi-scale high-level semantic features and low-level details, so as to achieve effective cross-layer feature fusion, suppress noise, and improve model segmentation performance. Experimental results show that Swin-Net performs significantly better than the most advanced methods on the two public benchmark datasets. In particular, it achieves an absolute improvement of 1.4–1.8% on Dice. Additionally, we provide a new dataset of breast ultrasound images on which we test the effect of our model, further demonstrating the validity of our method. In summary, the proposed Swin-Net framework makes significant advancements in breast ultrasound image segmentation, providing valuable exploration for research and applications in this domain.

## 1. Introduction

Breast cancer is the phenomenon that mammary epithelial cells proliferate out of control under the action of a variety of carcinogenic factors, and its incidence ranks first among female malignant tumors [1]. Clinical experience has shown that although the causes of breast cancer are not fully understood, early diagnosis is particularly important for treatment. At the clinical stage, the diagnostic methods of breast cancer are mainly divided into invasive diagnosis and non-invasive diagnosis [2]. The invasive diagnostic is also called biopsy. It is the ultimate basis for the diagnosis of breast cancer, but brings certain physiological trauma to patients. So, it is not necessary in most cases except malignancies [3]. Non-invasive diagnosis is the use of ultrasound imaging, X-ray imaging, nuclear magnetic resonance imaging, and other methods to carry out medical imaging examination of breast lesions. Compared with ultrasound imaging, the ionizing radiation produced by X-rays is harmful to patients and doctors. Although MRI imaging quality is better, the cost is higher. Ultrasound imaging is noninvasive and low cost, so it is the first choice for breast cancer diagnosis.

Ultrasound screening mainly relies on doctors’ observation of ultrasound images in the early diagnosis of breast cancer. However, the accuracy of diagnosis is affected by many uncontrollable realistic interference factors, which makes the diagnosis result prone to errors. Therefore, the clinical experience requirements for doctors are very high. To solve this problem, researchers introduced Computer Aided Diagnosis (CAD) to the diagnostic task of breast ultrasound images. In the assisted diagnosis of breast ultrasound images, the general workflow of CAD system is as follows: firstly, accurate segmentation of breast tumor region is carried out; then, feature extraction is carried out on the segmentation results; and finally, based on the extracted features, the classification of breast tumors is carried out using classifiers to judge the benign and malignant tumors [4]. The feature extraction should be carried out based on the image segmentation results, and the extracted features will directly affect the diagnostic results. Therefore, ultrasonic breast segmentation is a very key step in the CAD system and has important clinical significance.

**Figure 1:**
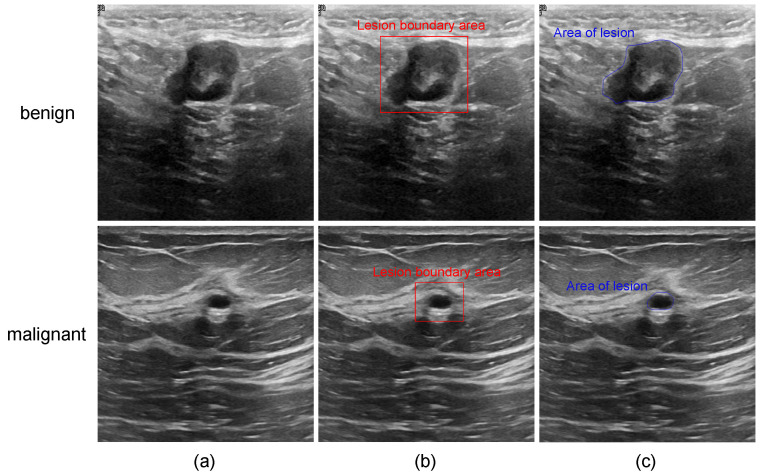
Some examples of breast ultrasound image: (**a**) original images of benign and malignant lesions; (**b**) images with the boundary of the lesion area; and (**c**) images with the specific lesion area.

Traditional image segmentation methods mainly rely on low-level features such as texture and geometric features [5]. Their segmentation quality is usually low, and their generalization is poor. With the development of deep learning technology, its role in the medical field is gradually emerging. In view of the defects of traditional algorithms, many scholars have begun to study the use of deep learning methods to further improve the accuracy of computer-aided systems for ultrasound breast tumor segmentation. Later, the U-Net [6] network framework was proposed. Many variant models based on U-net have been widely used in breast tumor segmentation due to its ability to utilize multi-level feature reconstruction to obtain high-resolution prediction results. Although the accuracy and generalization ability of these methods have been greatly improved compared with traditional methods, the accurate localization of tumor lesions still has certain limitations. Firstly, due to the inherent locality of the accepting domain in the convolution operation, various models based on this method cannot model the remote dependency explicitly. This often results in the inability to achieve optimal segmentation when capturing rich anatomical features of different shapes and scales (e.g., tumor regions with different structures and sizes). Moreover, due to the acquisition and imaging method of ultrasonic data and the possible improper operation in the acquisition process, ultrasonic imaging is characterized by large speckle noise, blurred region, weak boundary, low contrast, and difficulty in locating ROI, as shown in Figure 1, which greatly increases the difficulty of tumor detection.

To address the above issues, our contribution in this paper is threefold:

1. We propose a new framework for breast ultrasound segmentation, named Swin-Net. Unlike the existing CNN-based method, we use the swin-transformer with better encoding performance as our encoder.
2. To support the framework presented in this article, we use two simple modules to improve segmentation capabilities. Firstly, a feature refinement and enhancement module (RLM) is designed to further refine and enhance the feature map learned by the transformer encoder. Thus, the model can capture global information over long distances and learn more detailed information at the same time. Then, we developed an HFM module, which classifies the tumor region features with high-level semantic location information and pixel information with more underlying segmentation details, respectively, so as to achieve the effective fusion of cross-layer features in a specific way, thus effectively suppressing noise and improving segmentation accuracy.
3. Finally, we conducted extensive experiments on two challenging benchmark datasets BUSIS [7] and BUS-B [8], as well as BUS-O, the dataset we provided, to evaluate the performance of the proposed Swin-Net. On BUSIS, the Dice of our method reaches 0.818, 1.4% higher than that of the existing most advanced method, NU-Net [9]. On the BUS-B dataset, the Dice of our model is 0.837, 1.8% higher than that of NU-Net. On BUS-O, our method reaches 0.840 of Dice, 1.5% higher than NU-Net.

## 2. Related Works

### 2.1. Traditional Methods of Breast Tumor Segmentation

Traditional methods for medical ultrasound image segmentation are mainly based on a clustering algorithm, which involves unsupervised learning, that is, no manual labeling of the training set; it includes methods based on threshold, based on region growing, and based on active contour model [10].

Although these methods can simply segment the breast tumor region, they usually rely on the superficial morphological features of breast ultrasound images. So, they have limited segmentation capabilities and require human intervention. For example, the method based on threshold segmentation needs to manually select the threshold according to the gray value distribution. The method based on region growing needs to set the seed points manually. The method based on active contour then needs to give the initial contour artificially. Some methods improved on the traditional methods even without manual intervention to achieve automatic tumor segmentation but also need to carry out very complex pre-processing of the image. They are mostly composed of a variety of specific algorithms, which leads to the results being dependent on the specific data set, and the generalization of the algorithm is poor.

### 2.2. Breast Tumor Segmentation Methods Based on CNNs

In view of the shortcomings of traditional algorithms, many scholars have begun to study the use of deep learning methods for breast tumor segmentation. Yap et al. [11] propose an ultrasound breast tumor segmentation model using a convolutional neural network based on LeNet, and its segmentation results are significantly better than the traditional solutions, which proves the effectiveness of deep learning methods. FCN [12] pioneers the end-to-end fully convolutional neural network for semantic segmentation, and Yap et al. [13] adopt a method based on this framework to realize the segmentation of breast tumors. However, this method cannot solve the problem of tumor size and shape variability, resulting in unsatisfactory results. Since then, PSPNet [14], SegNet [15], U-Net [6], ResU-Net [16], Deeplab Series [17,18,19,20], U-net ++ [21], ResUNet++ [22], and other semantic segmentation networks have emerged one after another. Because the training of the above model requires more data, and the current public breast tumor segmentation datasets of ultrasound images are very limited, the network structure based on U-Net usually performs well when the amount of data is limited, so it is widely used in medical image segmentation tasks including breast tumor segmentation.

Zhuang et al. [23] propose an improved U-Net segmentation network RDAU-net for breast tumor segmentation in ultrasound images. Based on the traditional U-Net structure, the residual unit [16] is used to replace the ordinary neural unit. At the same time, atrous convolution is introduced in the encoder stage of U-Net, and the attention gate module [24] is used to replace the original clipping and copying operations in order to increase the network receptive field, suppress background information, and enhance the learning ability of the model. Punn and Agarwal [25] introduce a lightweight RCA-IUnet model guided by residual cross-spatial attention, which also follows the U-Net topology. The depthwise separable convolution [26], cross-spatial attention filter, and a hybrid pooling method are used to further improve the performance of tumor segmentation of different sizes in breast ultrasound images, on the basis of guaranteeing the segmentation ability. Lou et al. [27] develop a new multi-level context refinement network model (MCRNet) to achieve fully automatic semantic segmentation in ultrasound imaging. The feature fusion scheme of the U-net network is improved by adaptively reducing the semantic gap and enhancing the context relationship between the encoder and decoder features. Chen et al. [9] propose a nested U-net structure (NU-net) for the accurate segmentation of breast tumors. U-Net with different depths and shared weights is utilized to achieve a robust representation of breast tumors. Lyu et al. [28] proposed a breast ultrasound image segmentation model named AMS-PAN. The encoder part uses depthwise separable convolution to obtain multi-scale breast feature maps and combines the attention mechanism to achieve the effective segmentation of lesion regions. Iqbal and Sharif [29] propose an improved semi-supervised learning method (PDF-UNet) based on UNet, combining a data expansion network (DEN), a probability map generator network (PMG), and a U- shaped pyramid-dilated fusion network for breast tumor segmentation. Firstly, the DEN network is used to generate synthetic images for the data expansion task. Secondly, the PMG network is used to generate the corresponding probability map image for the synthesized unannotated image. Finally, they use the PDF-UNet network to segment breast tumor images.

### 2.3. Transformer Methods in Semantic Segmentation Tasks

Convolutional Neural Networks (CNNs) have become the de facto standard for medical image analysis tasks. However, with the continuous development of Transformer, especially its research and application in CV field, using Transformer has proved to be more promising in utilizing remote dependency in computer vision than other traditional CNN-based approaches [30]. At the same time, Transformer, with its strong global relationship modeling capabilities, has become the new standard starting point for a wide range of downstream training.

**Figure 2:**
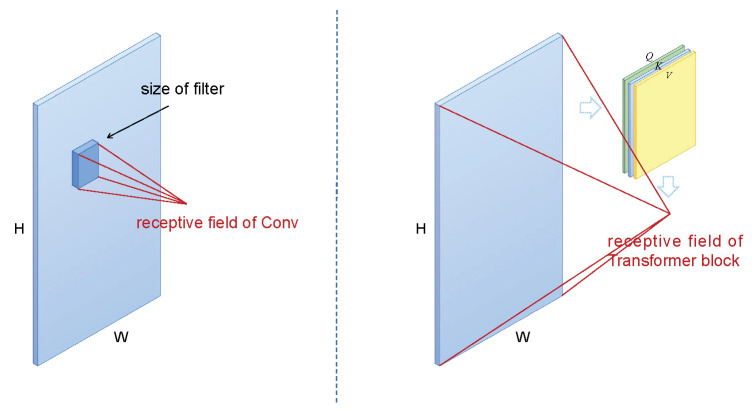
The rough illustration of the receptive field of CNN and the receptive field of Transformer.

Transformer was first proposed by Vaswani et al. [31] for machine translation tasks and has since had a wide impact in the field of natural language processing. Different from CNNs, Transformer uses the multi-head self-attention layer (MHSA) to model long-term dependencies. The MHSA layer has dynamic weights and global receptive field, as shown in the Figure 2, which makes the Transformer model more flexible and effective. To apply transformers to computer vision tasks, Dosovitskiy et al. [32] proposed a vision transformer (Vit), the first pure transformer architecture for image classification. The Vit model splits an image into multiple patches, encodes them, and sends them sequentially to the transformer encoder, which then uses MLP for image classification. However, the scale of images varies greatly and is not standard fixed, so the performance of Vit may not be good in different scenarios, and it is difficult to transplant Vit to various dense prediction tasks. At the same time, compared with text information, these pictures have larger pixel resolution. Due to the multi-head self-attention mechanism of Transformer, its computational complexity is the square of the number of tokens. Therefore, Liu et al. [33] proposed a hierarchical vision transformer using shifting windows (swin-transformer), which processes images through a hierarchical structure similar to CNNs, so that the model can flexibly process images of different scales. And window self-attention is used to reduce the computational complexity and process images of different scales. This shows that the swin-transformer architecture can easily replace the backbone network in existing methods for various vision tasks. In this work, we design a novel swin-transformer-based segmentation framework to achieve more accurate localization of breast tumor lesions in ultrasound images with typical noise interference.

In general, the current methods exhibit significant limitations, including:

- Inadequate adaptability for capturing both global and detailed information: Traditional methods face constraints due to their limited analysis of shallow features. CNN-based approaches struggle to effectively capture remote dependencies, while Transformer technology falls short in capturing detailed information.
- Limitations associated with the available training data: Deep learning models often demand extensive data for training. However, the availability of breast ultrasound datasets is limited.
- Insufficient segmentation performance: Breast ultrasound images pose challenges such as speckle noise, blurry boundaries, and low contrast, making tumor segmentation more challenging.

## 3. Methods

### 3.1. Overall Architecture

**Figure 3:**
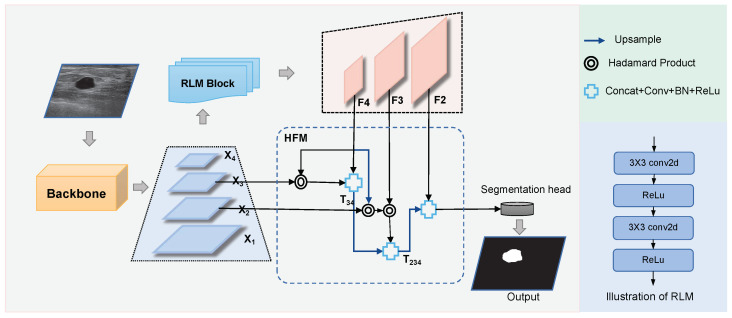
The illustration of Swin-Net, composed of swin-transformer encoder (Swin-T), feature refinement and enhancement module (RLM), and hierarchical multi-scale feature fusion module (HFM).

As shown in Figure 3, the proposed Swin-Net consists of three key modules: the swin-transformer encoder (Swin-T), the feature refinement enhancement module (RLM), the and hierarchical multi-scale feature fusion module (HFM). Specifically, the swin-transformer encoder is used to extract multi-scale long-range dependent features from the input image. The RLM algorithm is used to remove the influence of noise and to refine and enhance the feature representation information of breast nodules, including texture and edge. For feature maps of different scales, the low-level features contain more high-resolution detailed information, while the high-level feature maps contain more general semantic information. Therefore, HFM is designed to hierarchically fuse the different level feature maps processed by the RLM module, which can collect semantic clues and locate the lesion area by gradually aggregating the high-level features. Finally, the pixel-level information is effectively transmitted to the whole lesion area. Given an image of size H×W×3, we first divide it into small pieces of size 4×4. The transformer encoder learns multi-level image features $\left\{X_{1},X_{2},X_{3},X_{4}\right\}$ with multi-scale and different resolution granularity through Swin-T from the input original image. These dimensions of these features are $\left\{1/4,1/8,1/16,1/32\right\}$ of the original image resolution, respectively.

We then pass the features $\left\{X_{2},X_{3},X_{4}\right\}$ of the last three levels to the RLM module for further refinement enhancement learning of each feature map. We can obtain a hardened multi-level feature map $\left\{F_{2},F_{3},F_{4}\right\}$, which is fused by the HFM module to obtain a fusion-decoded feature map $O\in R^{H/4\times W/4\times 16}$. Finally, *O* is fed to the final segmentation header, mainly a convolutional layer of 1×1, to predict the segmentation mask at h×w×Ncls resolution. Ncls is a class number of 2, and the final prediction mask is constrained by the segmentation loss. In the rest of this section, we will detail the design of each proposed module and summarize the main differences between our approach and others.

### 3.2. Swin-Transformer Encoder

**Figure 4:**
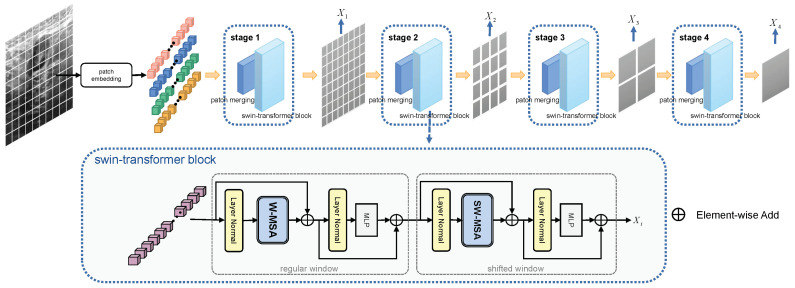
The illustration of Swin-T.

Due to hardware limitations and uncontrollable factors in the imaging process, breast ultrasound images often contain severe speckle noise. Several recent studies have found that vision transformers exhibit superior performance and better robustness to perturbations than CNNs. Inspired by this, we use a visual transformer as our backbone network to extract more robust and powerful features for breast ultrasound segmentation. Unlike Vit, which uses a fixed token size and a fixed columnar structure, Swin-T is able to build hierarchical feature maps, and its computational complexity scales linearly with image size. Multi-scale features captured in different Windows can provide information at different perceptual scales, which conforms to the characteristics of pixel-level dense prediction in visual tasks of semantic segmentation. At the same time, this can reduce resource consumption because the self-attention calculation is performed only within the window. Specifically, we adopt the Swin-TS [33], a lightweight version of Swin-T, which is more resource-efficient while having powerful feature extraction capabilities. In order to adapt Swin-T to the breast cancer ultrasound segmentation task, as shown in Figure 4, we remove the last classification layer to generate four multi-scale feature maps $\left\{X_{1},X_{2},X_{3},X_{4}\right\}$ at different stages. Among them, $\left\{X_{2}\right\}$ provides detailed appearance detail information of breast cancer lesions, and $\left\{X_{3},X_{4}\right\}$ provide high-level semantic features. Moreover, we design an ultrasound segmentation head on the feature maps of the last three levels.

### 3.3. Feature Refinement and Enhancement Module (RLM)

Transformer encoders have advantages in directly modeling global semantic interactions and contextual information, while CNNs have advantages in terms of spatial location representation and the learning of local spatial correlations. Therefore, for the multi-level feature map obtained in the encoder part, we refine the enhancement learning through the CNN-based RLM, which can further extract the fine-grained features to form the enhanced features and reduce the noise influence. Unlike common decoders that aggregate the features from stages 1 to 4, our decoder only receives features from the last three stages. This is due to the rendering characteristics of ultrasound images: the feature map of stage 1 often contains too much noise interference, which can impair performance. In addition, operations on $X_{1}$ introduce significant computational overhead. Therefore, we only perform RLM enhancement for the features of the latter three stages. The implementation method can be expressed as Equation (1)

(1) $$F_{i}=proj\left(f_{i}\left(X_{i}\right)\right)$$

(2) $$proj\left(x_{i}\right)=ReLU\left(Conv\left(ReLU\left(Conv\left(x_{i}\right)\right)\right)\right)$$

where $F_{I}$ is the enhanced feature after refinement, and $X_{i}$ is initial feature maps of the last three stages learned by the encoder, $\left(i=2,3,4\right)$. $f_{i}\left(\cdot \right)$ represents the upsampling function of the corresponding dimension of each stage feature map, and the bilinear interpolation algorithm is adopted. $proj\left(\cdot \right)$ is a refinement enhancement function consisting of two 1×1 convolutional layers and a ReLU activation function.

### 3.4. Hierarchical Multi-Scale Feature Fusion Module (HFM)

To make full use of features from different semantic levels, including original multi-scale features and enhanced multi-scale features, we design a hierarchical multiscale feature fusion module (HFM). As shown in Figure 3, we adopt different cascading processing mechanisms for different levels of high-level semantics and low-level details. The HFM gradually guides high-resolution low-level feature maps with low-resolution high-level semantic feature maps to achieve effective multi-scale feature fusion and improve the final segmentation performance.

We first fuse the highest-level feature map $F_{4}$ with the original feature map $X_{3}$ and use $L_{3}\left(\cdot \right)$ to smoothly connect the fused features to obtain the fusion feature map $T_{34}\in R^{H/16\times W/16\times 32}$. Here, *T* represents the fusion feature, and the subscript index is composed of the network layer numbers from which the features originated before fusion. For example, $T_{34}$ denotes the high-level fusion feature map obtained by fusing primary and refined features from the 4th and 3rd layers of the network. The process can be summarized as Equation (3).

(3) $$T_{34}=L_{3}\left(cat\left(F_{4}*X_{3}\right),F_{4}\right)$$

where ∗ represents the Hadamard product; $cat\left(\cdot \right)$ is a splicing operation along the channel dimension; and $L_{i}\left(\cdot \right)$ is composed of a 1×1 convolutional layer, a BN layer, and an activation layer for smoothing and dimensionality reduction.

Then, we use a similar process to fuse the refinement features $F_{3}$, $F_{4}$; the initial encoding feature $X_{2}$; and the fusion feature $T_{34}$. First, we upsample $F_{4}$ and $T_{34}$ to the same size as $X_{2}$ and perform feature smoothing. We then multiply the smoothed $F_{4}$ and $F_{3}$ with $X_{2}$ and stack the resulting feature map with the smoothed $T_{34}$. Finally, we input the stacked feature map into the fusion function $L_{2}\left(\cdot \right)$ to obtain $T_{234}\in R^{H/8\times W/8\times 32}$. Here, $T_{234}$ represents the fusion feature map obtained by fusing the features from layers 2, 3, and 4 of the network. The process is shown in Equation (4).

(4) $$T_{234}=L_{2}\left(concat\left(G_{i}\left(F_{4}\right)*F_{3}*X_{2},G_{i}\left(T_{34}\right)\right)\right)$$

(5) $$G_{i}\left(x\right)=ReLU\left(BN\left(Conv\left(f_{i}\left(x\right)\right)\right)\right)$$

where $f_{i}\left(\cdot \right)$ represents the bilinear interpolation algorithm, and $G_{i}\left(\cdot \right)$ is mainly composed of an interpolation function and a 3×3 convolutional layer, which is used to adjust the size of the feature map and smooth it.

Since the deep convolutional neural network usually loses some potential detailed features, we fuse the aggregated feature $T_{234}$ with the refined low-level feature $F_{2}$. Finally, we obtain the output $O\in R^{H/4\times W/4\times 16}$ of the HFM:

(6) $$O=L_{1}\left(concat\left(G_{i}\left(T_{234}\right),F_{2}\right)\right)$$

### 3.5. Loss

The data of medical images is very rare, and the positive and negative samples are unbalanced. Considering these issues, we design a more effective loss function to achieve a better segmentation effect, which combines cross-entropy loss, Dice loss, and intersecting and union loss as the overall segmentation loss. Therefore, the loss function of Swin-Net can be expressed as:

(7) $$L_{all}=\lambda L_{ce}+L_{dice}+L_{iou}$$

where λ is the empirical coefficient; in this paper, we set λ=2. This makes the model pay more attention to mining the foreground area in the training process and ensures the stability of the model loss convergence while improving the ability of the model to deal with the severe imbalance of positive and negative samples.

## 4. Results and Discussion

### 4.1. Experiment Materials

In this paper, two widely used public breast ultrasound datasets and a private dataset provided by ourselves are used to evaluate the performance of the segmentation network.

BUSIS: The first breast ultrasound dataset is BUSIS, proposed by Al-Dhabyani et al. [7]. It contains 780 images of 600 female patients, including 210 malignant cases, 437 benign cases, and 133 normal cases.

BUS-B: The second breast ultrasound DataSet used in this paper is BUS-B, collected by Yap et al. [11]. Dataset B contains a total of 163 images, including 110 benign and 53 malignant cases.

BUS-O: The third breast ultrasound dataset used is BUS-O, collected by ourselves from Hunnan Cancer Hospital. In this study, we collect a dataset of 267 ultrasound images of breast lesions from 170 different women, of which 109 were malignant and 161 benign lesions. Each image comes with its corresponding segmentation ground truth, annotated by two professional medical experts. It is crucial to emphasize that our data have received ethical approval and undergone rigorous ethical processing to ensure compliance with research integrity and ethical standards.

### 4.2. Experiment Configuration

The model proposed in this paper is built, trained, and tested based on the PyTorch (1.7.0) deep learning framework. We split the training set and validation set at 7:3, and all images are reshaped to 448×448 before entering the network.

Additionally, we preprocess the data with five-fold augmentation, such as random horizontal flipping, random rotation, random blurring, and random noise addition. Random horizontal flipping and random rotation change the perspective of the images, enhancing the adaptability of the model to different angles. Random blurring simulates the slight blurriness present in actual ultrasound images, making the model more robust. The random noise helps the model better handle image disturbances typical in real-world scenarios. These preprocessing steps introduce diversity and complexity to the data, contributing to improving the generalization ability of the model.

We use the AdamW optimizer [34] in all our experiments. To train our Swin-Net, the initial learning rate is set to 1 × 10^−4^. Each model is trained for 200 epochs for all datasets. The bath size is set to 8.

### 4.3. Evaluation Metrics

In order to quantify and compare the performance of the proposed model and the contrast models in breast ultrasound image segmentation, we select five classic quantitative evaluation metrics as follows: Global Accuracy (Acc), Intersection over Union (Iou), DiceSimilarity Coefficient (Dice), precision, and recall. The higher the value of these five metrics, the better the segmentation effect of the network.

### 4.4. Comparison of Results

In this section, our model is compared with several open-source methods, including U-Net [6], Seg-Net [15], Unet++ [21], Att-Unet [24], Res-Unet++ [22], CE-Net [35], SK-UNet [36], CPF-Net [37], and Nu-Net [9]. Among them, U-net and Seg-net are classical models in the field of image segmentation, and Unet++, Att-U-net, and Res-Unet are effective improved variants of U-Net. CE-net, SK-Unet, and CPF-Net are advanced networks suitable for medical image segmentation tasks, and Nu-Net is the most advanced method for the ultrasound image segmentation of breast tumor. The results demonstrate the effectiveness of different components of Swin-Net. Finally, we comprehensively evaluate the robustness of our approach. For fair comparison, we use their open source code for evaluation on the same training and test sets.

#### 4.4.1. Comparison with the Most Advanced Methods

**Figure 5:**
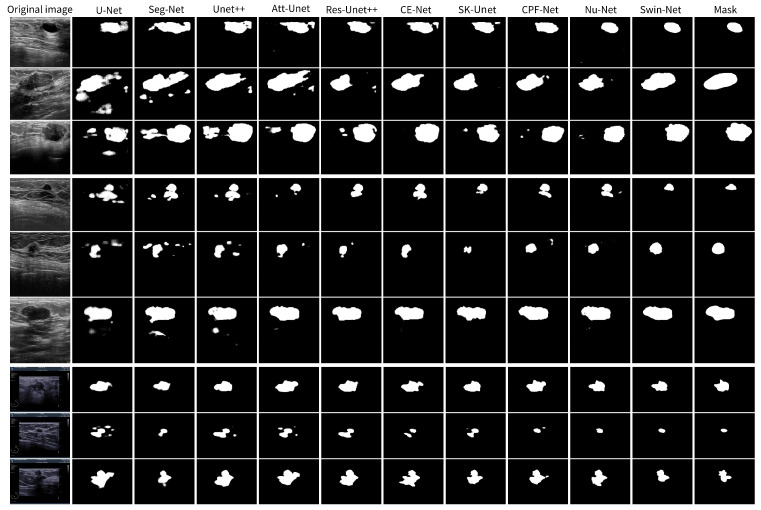
Qualitative comparison of various methods on different datasets. The first column is the original image; the last column is the ground truth; the penultimate column is our proposed Swin-Net segmentation prediction example; and the middle is the prediction result of the U-Net, Seg-Net, Att-Unet, Unet++, Res-Unet++, CE-Net, SK-Unet, CPF-Net, and Nu-Net models, respectively. Among them, the first three rows of images are from BUSIS, the middle three rows belong to BUS-B, and the last three rows are from BUS-O.

We compare the proposed method with nine advanced deep learning medical image segmentation methods. Table 1 shows the quantitative evaluation results of BUSIS via different methods, Table 2 shows the results of BUS-B, and Table 3 shows the results of BUS-O. The experimental results show that our method achieves the best segmentation performance on each dataset, especially achieving significant improvements of 1.4%, 1.8%, and 1.5% on the Dice index, respectively. In order to compare the segmentation performance more intuitively, we selected some representative examples from the test set of each dataset. As shown in Figure 5, the proposed Swin-Net can suppress irrelevant information more effectively, with clearer and smoother boundaries. Even when dealing with cases such as blurred boundaries and low contrast, our method can capture more precise quality regions, and the results can be closer to the annotations of experts.

#### 4.4.2. Ablation Study

To verify the effectiveness of the main components of Swin-Net: the Feature Refinement Enhancement Module (RLM) and the Hierarchical Multiscale Feature Fusion Module (HFM), we use the swin-transformer encoder as the baseline, comparing its variants with the standard version by removing or replacing components from the full Swin-Net. “Swin-Net(Swin-T+RLM+HFM)” represents the proposed version, and “RLM” and “HFM” indicate the usage of RLM and HFM, respectively. We perform ablation experiments on three datasets: BUSIS, BUS-B, and BUS-O. The effectiveness of our proposed methods can be seen from Table 4, Table 5 and Table 6.

Effectiveness of RLM: To analyze the effectiveness of RLM, we train a “swin-t(w/o RLM)” version by removing the RLM module from the entire Swin-Net. All evaluation metrics of the model without RLM have significant decreases on all three datasets compared to standard swin-PVT. In particular, Table 5 showes that Dice decreases from 0.840 to 0.813 on BUS-O.

Effectiveness of HFM: Similarly, we remove the HEM module from Swin-Net and replace it with a classic All-MLP Decoder (called “swin-t(w/o HFM)” to test its effectiveness. As shown in Table 4, Dice and Iou of full Swin-Net improve by 2.5% and 2.2% on BUSIS, respectively.

The validity of feature map selection: To verify the effectiveness of our method in feature map selection, we train the “swin-t(w/o X1)” version, which indicates that the output of stage 1 is also sent to the decoder. As shown in Table 4, Table 5 and Table 6, compared with Swin-Net, all evaluation metrics decrease on the three datasets. The results prove that the feature map of stage 1 is more disturbed by noise, which will affect the performance of the network.

## 5. Conclusions

In this paper, we introduce Swin-Net, an effective segmentation framework combining CNNs and Transformer for 2D breast tumor ultrasound image segmentation. Its key insight is to use swin-transformer as an encoder to obtain multi-level pyramid structure feature maps, which contain rich global spatial information and local multi-scale context information. In addition, Swin-Net also benefits from the feature refinement and enhancement module (RLM) proposed by us according to the characteristics of ultrasonic image. Combined with the advantages of CNNs, it can further refine the initial encoded feature map to achieve feature enhancement. Finally, a hierarchical multi-scale feature fusion module (HFM) is designed to realize the effective fusion of feature maps containing different semantic levels in stages and improve the segmentation performance. The experimental results indicate that Swin-Net performs competitively on two widely used breast ultrasound datasets and our proprietary dataset. Quantitatively, our Swin-Net outperforms the state-of-the-art Nu-Net on key metrics across multiple datasets. On the BUSIS dataset, we achieve higher values in Acc, IoU, Dice, precision, and recall. Similar superior performance is observed on the BUS-B and BUS-O datasets. Specifically, on BUSIS, Swin-Net achieves Acc of 0.959, IoU of 0.738, Dice of 0.818, precision of 0.834, and recall of 0.844, surpassing Nu-Net by approximately 0.42% 1.38%, 1.74%, 1.92%, and 0.72%, respectively. In addition to the experimental results, we have conducted a comprehensive analysis to assess the robustness of our approach. However, our study is still constrained by data limitations, and a more diverse and comprehensive dataset could contribute to improving the generalization ability of the model. Accurate ultrasound segmentation of breast tumors remains a challenge, and the segmentation performance may be affected in more complex pathological cases, necessitating further research. We believe that exploring effective data augmentation and preprocessing methods could enhance the robustness of the model to ultrasound image characteristics, particularly in addressing issues such as combating speckle noise. Additionally, considering the integration of information from other modalities of medical imaging, such as MRI or CT, may enhance accuracy in segmenting different types of lesions. In conclusion, we hope that our study will inspire new ideas to solve the problem of ultrasonic segmentation of breast tumors and contribute to the ambitious goal of building safer and more trustworthy clinical AI.

## Figures and Tables

**Table 1:** Comparison of segmentation results of benign and malignant breast lesions on dataset BUSIS by different methods. (Bold text indicates the best result).

	Train			Test		
Methods	Acc	Acc	Iou	Dice	Precision	Recall
U-Net [6]	0.979	0.915	0.569	0.629	0.642	0.681
Seg-Net [15]	0.988	0.922	0.621	0.703	0.708	0.723
Unet++ [21]	0.982	0.930	0.625	0.714	0.741	0.720
Att-Unet [24]	0.975	0.931	0.657	0.718	0.757	0.732
Res-Unet++ [22]	0.984	0.930	0.654	0.721	0.749	0.740
CE-Net [35]	0.992	0.943	0.671	0.723	0.785	0.765
SK-UNet [36]	0.986	0.945	0.682	0.753	0.799	0.762
CPF-Net [37]	0.979	0.952	0.691	0.776	0.824	0.796
Nu-Net [9]	0.988	0.955	0.725	0.804	0.818	0.838
Swin-Net	**0.994**	**0.959**	**0.738**	**0.818**	**0.834**	**0.844**

**Table 2:** Comparison of segmentation results of benign and malignant breast lesions on dataset BUS-B by different methods. (Bold text indicates the best result).

	Train			Test		
Methods	Acc	Acc	Iou	Dice	Precision	Recall
U-Net [6]	0.975	0.918	0.615	0.655	0.683	0.733
Seg-Net [15]	0.984	0.921	0.641	0.708	0.725	0.724
Unet++ [21]	0.978	0.926	0.622	0.726	0.742	0.781
Att-Unet [24]	0.984	0.927	0.653	0.753	0.759	0.762
Res-Unet++ [22]	0.987	0.939	0.671	0.759	0.756	0.774
CE-Net [35]	0.991	0.947	0.696	0.783	0.787	0.798
SK-UNet [36]	0.993	0.956	0.698	0.785	0.817	0.808
CPF-Net [37]	0.985	0.961	0.704	0.796	0.824	0.839
Nu-Net [9]	0.994	0.967	0.738	0.819	0.837	0.854
Swin-Net	**0.997**	**0.971**	**0.745**	**0.837**	**0.856**	**0.863**

**Table 3:** Comparison of segmentation results of benign and malignant breast lesions on dataset BUS-O by different methods. (Bold text indicates the best result).

	Train			Test		
Methods	Acc	Acc	Iou	Dice	Precision	Recall
U-Net [6]	0.971	0.919	0.638	0.704	0.710	0.691
Seg-Net [15]	0.982	0.920	0.657	0.714	0.709	0.729
U-Net++ [21]	0.987	0.922	0.696	0.726	0.717	0.743
Att-Unet [24]	0.973	0.931	0.716	0.753	0.756	0.770
Res-Unet++ [22]	0.996	0.935	0.704	0.767	0.759	0.794
CE-Net [35]	0.985	0.938	0.728	0.782	0.778	0.817
SK-UNet [36]	0.991	0.945	0.713	0.799	0.780	0.823
CPF-Net [37]	0.988	0.943	0.744	0.816	0.828	0.830
Nu-Net [9]	0.998	0.954	0.749	0.825	0.835	**0.853**
Swin-Net	**0.999**	**0.963**	**0.754**	**0.840**	**0.855**	0.850

**Table 4:** Ablation study on BUSIS. (Bold text indicates the best result).

	Train			Test		
Methods	Acc	Acc	Iou	Dice	Precision	Recall
swin-t(w/o RLM)	0.991	0.947	0.708	0.795	0.817	0.821
swin-t(w/o HFM)	0.990	0.955	0.716	0.793	0.809	0.832
swin-t(w/o X1)	0.989	0.946	0.724	0.805	0.818	0.838
Swin-Net(Swin-T+RLM+HFM)	**0.994**	**0.959**	**0.738**	**0.818**	**0.834**	**0.844**

**Table 5:** Ablation study on BUS-B. (Bold text indicates the best result).

	Train			Test		
Methods	Acc	Acc	Iou	Dice	Precision	Recall
swin-t(w/o RLM)	0.991	0.964	0.726	0.823	0.845	0.840
swin-t(w/o HFM)	**0.997**	0.965	0.732	0.827	0.855	0.845
swin-t(w/o X1)	0.987	0.961	0.724	0.820	0.832	0.856
Swin-Net(Swin-T+RLM+HFM)	**0.997**	**0.971**	**0.745**	**0.837**	**0.856**	**0.863**

**Table 6:** Ablation study on BUS-O. (Bold text indicates the best result).

	Train			Test		
Methods	Acc	Acc	Iou	Dice	Precision	Recall
swin-t(w/o RLM)	0.995	0.954	0.740	0.813	0.824	0.818
swin-t(w/o HFM)	**0.999**	0.955	0.733	0.818	0.844	0.816
swin-t(w/o X1)	0.998	0.953	0.746	0.826	0.866	0.835
Swin-Net(Swin-T+RLM+HFM)	**0.999**	**0.963**	**0.754**	**0.840**	**0.855**	**0.850**

## Data Availability

The data presented in this study are available on request from the corresponding author. The data are not publicly available due to privacy.
